# The koala gut microbiome is largely unaffected by host translocation but rather influences host diet

**DOI:** 10.3389/fmicb.2023.1085090

**Published:** 2023-03-02

**Authors:** Michaela D. J. Blyton, Jack Pascoe, Emily Hynes, Rochelle M. Soo, Philip Hugenholtz, Ben D. Moore

**Affiliations:** ^1^Hawkesbury Institute for the Environment, Western Sydney University, Richmond, NSW, Australia; ^2^The University of Queensland, Australian Institute of Bioengineering and Nanotechnology, St Lucia, QLD, Australia; ^3^Conservation Ecology Centre, Cape Otway, VIC, Australia; ^4^School of Ecosystem and Forest Science, University of Melbourne, Parkville, VIC, Australia; ^5^Ecoplan Australia, Torquay, VIC, Australia; ^6^The University of Queensland, School of Chemistry and Molecular Biosciences, Australian Centre for Ecogenomics, St Lucia, QLD, Australia

**Keywords:** microbial community, body condition, *Phascolarctos cinereus*, marsupial, eucalypt, hindgut fermenter

## Abstract

**Introduction:**

Translocation is a valuable and increasingly used strategy for the management of both threatened and overabundant wildlife populations. However, in some instances the translocated animals fail to thrive. Differences in diet between the source and destination areas may contribute to poor translocation outcomes, which could conceivably be exacerbated if the animals’ microbiomes are unsuited to the new diet and cannot adapt.

**Methods:**

In this study we tracked how the faecal microbiome of a specialist *Eucalyptus* folivore, the koala (*Phascolarctos cinereus*), changed over the course of a year after translocation. We assessed microbiome composition by 16S rRNA amplicon sequencing of faecal pellets.

**Results:**

We found no significant overall changes in the faecal microbiomes of koalas post-translocation (*n *= 17) in terms of microbial richness, diversity or composition when compared to the faecal microbiomes of koalas from an untranslocated control group (*n *= 12). This was despite the translocated koalas feeding on a greater variety of *Eucalyptus* species after translocation. Furthermore, while differences between koalas accounted for half of the microbiome variation, estimated diets at the time of sampling only accounted for 5% of the variation in the koala microbiomes between sampling periods. By contrast, we observed that the composition of koala faecal microbiomes at the time of translocation accounted for 37% of between koala variation in post-translocation diet. We also observed that translocated koalas lost body condition during the first month post-translocation and that the composition of the koalas’ initial microbiomes were associated with the magnitude of that change.

**Discussion:**

These findings suggest that the koala gut microbiome was largely unaffected by dietary change and support previous findings suggesting that the koala gut microbiome influences the tree species chosen for feeding. They further indicate that future research is needed to establish whether the koalas’ gut microbiomes are directly influencing their health and condition or whether aspects of the koala gut microbiomes are an indicator of underlying physiological differences or pathologies. Our study provides insights into how animal microbiomes may not always be affected by the extreme upheaval of translocation and highlights that responses may be host species-specific. We also provide recommendations to improve the success of koala translocations in the future.

## Introduction

Anthropogenic habitat loss, resource decline, climate change and ecological imbalances such as overpopulation threaten many mammal populations. One practical strategy available to environmental managers to promptly alleviate these threats is to translocate animals (Fischer and Lindenmayer, 2000; Menkhorst et al., 2019). However, in some instances the translocated animals fail to thrive and have higher rates of mortality than those left *in situ* (Fischer and Lindenmayer, 2000; Whisson et al., 2012; Menkhorst, 2017). These higher rates of mortality may in part be due to factors such as stress from capture and transportation, a lack of familiarity with the location of local resources such as denning sites, inexperience with local predators, a loss of territory and social structure and competition with resident conspecifics (Fischer and Lindenmayer, 2000; Massei et al., 2010). There are also a range of other, less studied, reasons for high rates of mortality in translocated animals including differences between the source and destination habitats. For instance, if the diet available to the animals at the destination location is different from that at the source, then translocated animals may have reduced fitness post translocation. This is especially likely to be true for specialist herbivores that rely on their gut microbiome to digest and detoxify otherwise unpalatable material, particularly if the animals’ microbiomes are unsuited to the new diet and cannot rapidly adapt (Kohl et al., 2014). Alternatively, in instances where a range of food types are available in the destination habitat and the gut microbiome of the translocated species has been shown to influence diet (Blyton et al., 2019), it is conceivable that the hosts’ microbiome may contribute to diet selection and habitat use post-translocation. In this study we track how the fecal microbiome of a specialist arboreal folivore, the koala (*Phascolarctos cinereus*), changes over the course of a year post translocation and investigate the associations between fecal microbiome composition, diet and host post-translocation condition.

Koalas are listed as endangered in the Australian states of Queensland and New South Wales as well as the Australian Capital Territory (Department of Agriculture, W.a.t.E., Australian Federal Government, 2022), where many populations are in decline. By contrast, in several areas of the southern Australian states of Victoria and South Australia, koalas can reach unsustainable population densities, resulting in the over-browsing of preferred food tree species, leading to habitat destruction and starvation (Martin, 1985a, 1985b, 1985c; Menkhorst, 2008; Whisson et al., 2016). In response, translocations of koalas away from high density populations into apparently suitable habitat with low koala densities plays a role in the ongoing management of koalas in these states (Menkhorst, 2008). Many of these translocations have been successful with high survival rates, while others where the destination habitat patch is small or located in agricultural land have had high rates of mortality (Whisson et al., 2012; Menkhorst, 2017). Koalas are also translocated throughout Australia to remove them from danger (unsuitable habitat) or where their habitat has been lost (Phillips, 2017). Further, translocations and breed-to-release programs are being developed to establish and expand threatened koala populations in north-eastern Australia.

Koalas are dietary specialists, feeding almost exclusively on eucalypt leaves, predominately from the genus *Eucalyptus* (Shipley et al., 2009). Koalas in different geographic areas feed on different species in part due to local availability. Additionally, individual koalas within populations can show preferences for different food tree species (Moore and Foley, 2000; Brice et al., 2019; Marsh et al., 2021) and koalas feeding on different species of *Eucalyptus* have functionally and compositionally different microbiomes (Brice et al., 2019). The koala gut microbiome also appears to influence what species of eucalypt the host can consume. This was tested in an experiment where koalas that primarily fed on *Eucalyptus viminalis* in the wild were inoculated with fecal material from donor koalas that fed primarily on *Eucalyptus obliqua.* The microbiomes of treatment koalas changed to resemble those of the donor koalas while those of control koalas did not, and the degree of change was associated with the amount of *E. obliqua* that koalas subsequently ate (Blyton et al., 2019). Thus, in the case of translocations, if the food tree species present at the destination habitat differ from those in the source habitat, then koala gut microbiomes may reduce the likelihood of good health and survival by limiting their hosts’ ability to feed and obtain nourishment from the new potential food species, unless the gut microbiomes can change and adapt to the new diet.

Across a range of other species with varied gastrointestinal anatomies and diets, including Tasmanian devils, Pere David’s deer, giant pandas and Atlantic salmon, the fecal microbiomes of individuals translocated between geographic areas, or released from captivity, change to resemble those of resident animals at the destination site (Chong et al., 2019; Wang et al., 2019; Yao et al., 2019; Uren Webster et al., 2020). However, koalas are unusual as they inherit their gut microbiomes maternally by feeding on pap (special maternal feces that has a higher microbial density than normal feces and a higher abundance of rare taxa) around the time of pouch emergence (Osawa et al., 1993; Blyton et al., 2022a, 2022b). Wild adult koalas have temporally stable fecal microbiomes over the course of several months when they are left in their established home ranges, despite some variation in the food tree species eaten (Eisenhofer et al., 2022; Blyton et al., 2023). Additionally, in 2013 koalas in Cape Otway, Victoria, that fed on *E. viminalis* starved to death when that species was defoliated due to overbrowsing rather than feeding on the readily available *E. obliqua* (Whisson et al., 2016). We subsequently showed that the fecal microbiomes of koalas that fed on *E. viminalis* in the wild did not change when they were experimentally encouraged to eat some *E. obliqua* in captivity, unless fecal inoculations were provided (Blyton et al., 2019). This suggests that the koala gut microbiome may not readily change after host translocation even when the microbiome is unsuited to the diet available.

In this study we assessed: (1) whether the composition of koala fecal microbiomes shifted after translocation in comparison to those of control koalas, captured but released immediately at the site of capture at Cape Otway, Victoria; (2) whether the fecal microbiomes of the koalas prior to translocation predicted their post-translocation body condition (a measure of their resilience during translocation); and (3) whether the fecal microbiomes of the koalas prior to translocation predicted their diets in the destination habitat, to further examine the role of the koala gut microbiome in diet selection.

## Methods

### Study design and sampling

In September 2015, 60 koalas were included in a trial translocation study conducted by the Victorian State Government Department of Environment, Land, Water and Planning (Menkhorst et al., 2019). The aim of the study was to assess the health and survival outcomes for koalas after they were translocated into mixed eucalypt forest from an area exhibiting severe overbrowsing and koala starvation (Whisson et al., 2016). All koalas were captured using a standard noose and flag technique (Madani et al., 2020) from herb-rich woodlands canopied by manna gum (*Eucalyptus viminalis*) and messmate (*Eucalyptus obliqua*) at Cape Otway, Victoria, Australia (38.825°S, 143.525°E). The koalas were assessed by veterinarians and only healthy adult individuals were included in the study (see Menkhorst et al., 2019 for selection criteria and veterinary procedures). All females received a subcutaneous contraceptive implant containing levonorgestrel (a synthetic progesterone) to ameliorate the risk of overabundance at the release site (Middleton et al., 2003; Hynes et al., 2010). The koalas were fitted with collar-mounted VHF radio transmitters. Twenty-four koalas were assigned to the control group and were released at the point of capture. The remaining 36 koalas were translocated a distance of approximately 90 kilometers northeast and released into coastal mixed eucalypt forest near Aireys Inlet, Victoria. All koalas were radio tracked on a regular basis to determine their location and survival status. The koalas were re-captured 1 month and 5 months after their initial capture to assess their health and condition, including weight and head length. All VHF collars were to be removed 1 year post translocation, however, only 9 of the koalas included in this study (control: males = 3, females = 2; Translocated: males = 2, females = 2) were located and caught at that time. This was because the VHF signals from the collars were no longer being transmitted due to premature failure of the collars’ batteries.

To assess how the koalas’ gut microbiomes and diet changed after translocation, we collected as many fecal pellets (range:1–70) as available from each translocated and control koala located during each sampling period. The sampling periods were: (1) when the koalas were first captured (day 0, number of koalas = 29); (2) within the first 2 days post-translocation (days 1–2, koalas = 17); (3) 1 week post-translocation (days 6–8, koalas = 14); (4) 2 weeks post-translocation (days 14–16, koalas = 11); (5) 1 month post-translocation (days 25–36, koalas = 16); (6) 2 months post-translocation (days 62–70, koalas = 14); (7) 5 months post-translocation (days 129–161, koalas = 26); (8) 9 months post-translocation (days 253–281, koalas = 15; these samples were only used for diet analysis); and (9) 1 year post-translocation, at the conclusion of the study (days 345–356, koalas = 9 with one koala sampled on two occasions during this period). Fecal pellets were either collected opportunistically during koala captures or from plastic mats placed beneath radio-tracked koalas. Pellets were generally frozen and then stored at –20°C within 2 h of excretion and all were frozen within 10 h. It was not always possible to collect samples during each sampling period/time point for an animal as they often did not produce any pellets while researchers were present. Only animals for which an initial sample and at least one post-translocation sample were collected were included in our analyses. Following these criteria we included 17 translocated (female = 8, male = 9) and 12 control koalas (female = 6, male = 6) in our study. On average, samples were available from 4.7 time points per koala.

### Microbiome characterization and bioinformatics

We determined the microbiome composition of 137 fecal samples (29 koalas sampled on between 2 and 8 occasions across time points) using a culture-independent DNA based approach. For each fecal sample, total genomic DNA was extracted between May and November 2016 from approximately 50–70 mg of fecal material. The material was taken from the center of a single fecal pellet to avoid any surface contamination. The material was beaten for 5 min at 2,000 rpm using the MoBio PowerLyzer24 in a MoBio bead tube containing 0.1 mm dia. Zirconian/silica beads and 750ul of TLA buffer (Promega). The samples were centrifuged at 10,000 g for 30 s. DNA was then extracted from 150 μl of the supernatant using the Maxwell 16 robotic system and corresponding Tissue DNA kit (Promega) following the manufacturer’s instructions. Negative controls were included for each extraction kit.

A 589 bp section of the 16 s rRNA gene (V6 – V8 region) was amplified using 803F (5’-TTAGANACCCNNGTAGTC) and 1392R primers (5’-TTAGANACCCNNGTAGTC, Engelbrektson et al., 2010) from the DNA extracts following the workflow outlined by Illumina (#15044223 Rev.B) except that Q5 Hot Start High-Fidelity 2X Master Mix (New England Biolabs) was used. PCR products were indexed with unique 8 bp barcodes using the Illumina Nextera XT 384 sample Index Kit A-D (Illumina FC-131-1,002). Indexed amplicons were isolated using Qiagen QIAquick Gel Extraction Kit, as per manufacturer’s instructions. Paired-end sequencing was performed at the Australian Centre for Ecogenomics, on the Illumina Miseq using the version 3 reagent kit for 300 cycles within 6 months of DNA extraction. The raw sequencing data from the study can be obtained from the NCBI SRA database, BioProject accession PRJNA901215.

Raw reads were trimmed to remove primer sequences using Cutadapt (Martin, 2011), and quality trimmed to remove poor quality sequence using a sliding window of 4 bases with an average base quality above 15 using the software Trimmomatic (Bolger et al., 2014). All reads were then hard trimmed to 250 bases, and any with less than 250 bases excluded. Trimmed reads were then processed and assigned taxonomic designations by QIIME 2 with default parameters (v.2017.10; Bolyen et al., 2019) using the SILVA 128 database (Quast et al., 2012; Yilmaz et al., 2013). The resulting microbial feature-by-sample table was rarefied to 10,000 reads per sample using the vegan package, version 2.6–2 (Oksanen et al., 2022) in R (version 3.5.0; R Core Team, 2012). All community composition analyses were performed on the rarefied dataset. Microbiome richness was estimated by a count of unique features recovered per sample after rarefaction and by calculating the Chao Index of alpha diversity using the package fossil in R (Vavrek, 2011). Microbiome diversity for each koala at each timepoint was estimated using the Shannon diversity index as calculated using the vegan package. Weighted and unweighted Unifrac distances (Lozupone and Knight, 2005; Lozupone et al., 2007) between samples were calculated in QIIME 2 from a filtered rarefied sample-by-features table where only features with greater than 10 reads in at least one sample were included.

### Diet determination

Diet composition for the translocated koalas was characterized by amplifying and sequencing dietary DNA fragments containing species-specific SNPs from pooled DNA extractions of two fecal pellets (when available) from each of the fecal samples for which microbiome assessment was performed. This was done using the DarTag platform at Diversity Arrays Technologies (Blyton et al., 2023). Further information on the diet analysis method and details of the determination of diet composition for the koalas included in this study is provided in Blyton et al. (2023). To provide an overall assessment of the diet of each koala, the proportions of each of the food tree species were averaged across all samples collected after 1 week post translocation. Samples collected within the first week post translocation were excluded from this assessment as the koalas’ diets may have been heavily influenced by their release location. Koalas from the control group were not included in this analysis.

### Statistical analysis

#### Microbiome change in response to host translocation

To assess if the koalas’ fecal microbiomes changed in response to translocation, linear mixed effects models were fitted using the lme4 package in R (Bates et al., 2015) with statistical significance calculated using the lmerTest package (Kuznetsova et al., 2013). To assess if microbial richness or diversity changed in response to translocation, the counts of the microbial features for each koala at each sampling time point, the Chao1 Index of alpha diversity and the Shannon diversity index were fitted as response variables. To assess if the composition of the koala fecal microbiomes changed in response to translocation, the unweighted and weighted unifrac distances between the post translocation samples and the day 0 samples for each koala were fitted as response variables and whether the koalas were translocated included as a fixed explanatory variable. Three alternative temporal explanatory variables were considered along with translocation status in separate models. Firstly, the sampling time point was fitted as a factor to determine if there were any particular time points that were distinct. Secondly, the number of days since translocation was fitted as a continuous variable to model a gradual change in microbiome composition. Thirdly, log10 (number of days since translocation) was fitted as a continuous explanatory variable to account for the possibility that the fecal microbiomes had shifted rapidly after translocation and then stabilized. The interaction of Treatment × timepoint or interval since translocation was also included, while koala identity was included as a random effect to account for the repeated measures sampling design. Backward elimination of non-significant terms was performed. The assumption of normality of the model residuals was assessed by the Shapiro–Wilk test and where necessary the response variable rank-transformed to meet this assumption.

To assess whether the microbiomes of translocated koalas were more variable over time than those of control koalas, average unweighted and weighted unifrac distances between sampling time points were calculated for each koala. Linear models were then fitted to the rank-transformed average unifrac distances, with translocation status as the explanatory variable.

To assess whether the relative abundance of particular microbial features increased or decreased in the translocated or control koalas over time we fitted betabionomial mixed effect regression models using the glmmTMB package v.1.1.4 in R Core Team (2012). All features that were detected on at least 2 occasions within three koalas in each of the translocated and control groups of koalas were assessed (*n* = 132). The explanatory variables in the analysis were the number of days since translocation or log10 (number of days since translocation) and whether the koalas were translocated. The interaction of treatment x interval since translocation was also included, while koala identity was included as a random effect. Significance was determined from Bonferroni corrected *p*-values for multiple comparisons.

#### Effect of the gut microbiome on host body condition

Koala body condition was calculated from the residuals of a linear regression of koala head length against body weight according to the method of McLean (2003). Linear regression models were then fitted to the change in body condition over the first month post-translocation. The coordinates of the koalas’ initial microbiomes on the first 5 dimensions generated from the PCoAs of (1) weighted and (2) unweighted unifrac distances were fitted as explanatory variables along with (3) the Shannon Indices of the initial microbiomes; (4) the Chao Index of the initial microbiomes; (5) the proportions of each food tree species in the koalas’ diets averaged over time points; and (6) the first 2 principal coordinates of the koalas’ diets. Each set of explanatory variables (1–6) were fitted in separate models with backwards elimination used within each set to select significant predictors where appropriate.

#### Effect of the gut microbiome on diet

To assess if the composition of the koalas’ microbiomes influenced diet selection post-translocation, Redundancy Analysis was performed on the Bray-Curtis distances between the koalas’ overall (averaged post-translocation) diets using the vegan package in R. The explanatory variables in the analysis included all principal components generated from the weighted and unweighted unifrac distances between the translocated koalas’ microbiomes on day 0 as well as the number of microbial features for each koala on day 0, the Chao1 Index of alpha diversity and the Shannon diversity index of the koalas’ microbiomes on day 0. Forward and backward selection was used to select significant explanatory variables based on adjusted *p*-values.

To assess if the diversity of the koalas’ microbiomes influenced the diversity of species from which they fed in the destination habitat, linear regression models were fitted in R. The response variables were the number of tree species present in the diet and the Shannon index of dietary diversity. The explanatory variables were the Chao1 Index of alpha diversity and the Shannon diversity index of the koalas’ microbiomes on day 0.

#### Association between diet and microbiome variation

To assess if variation in the diets of translocated koalas was associated with temporal variation in fecal microbiomes, Redundancy Analysis was performed on the Bray-Curtis distances between koala microbiomes at all time points generated from the square root transformed proportions of the microbial features. The explanatory variables in the analysis included the proportion of each food tree species at each time point as well as the principal coordinates generated from the unscaled food tree species proportions. Koala ID was included as a covariate in this analysis to account for interindividual differences in fecal microbiome composition (Eisenhofer et al., 2022). Forward and backward selection was used to select significant explanatory variables based on adjusted p-values.

## Results

### Microbiome change in response to host translocation

**Figure 1:**
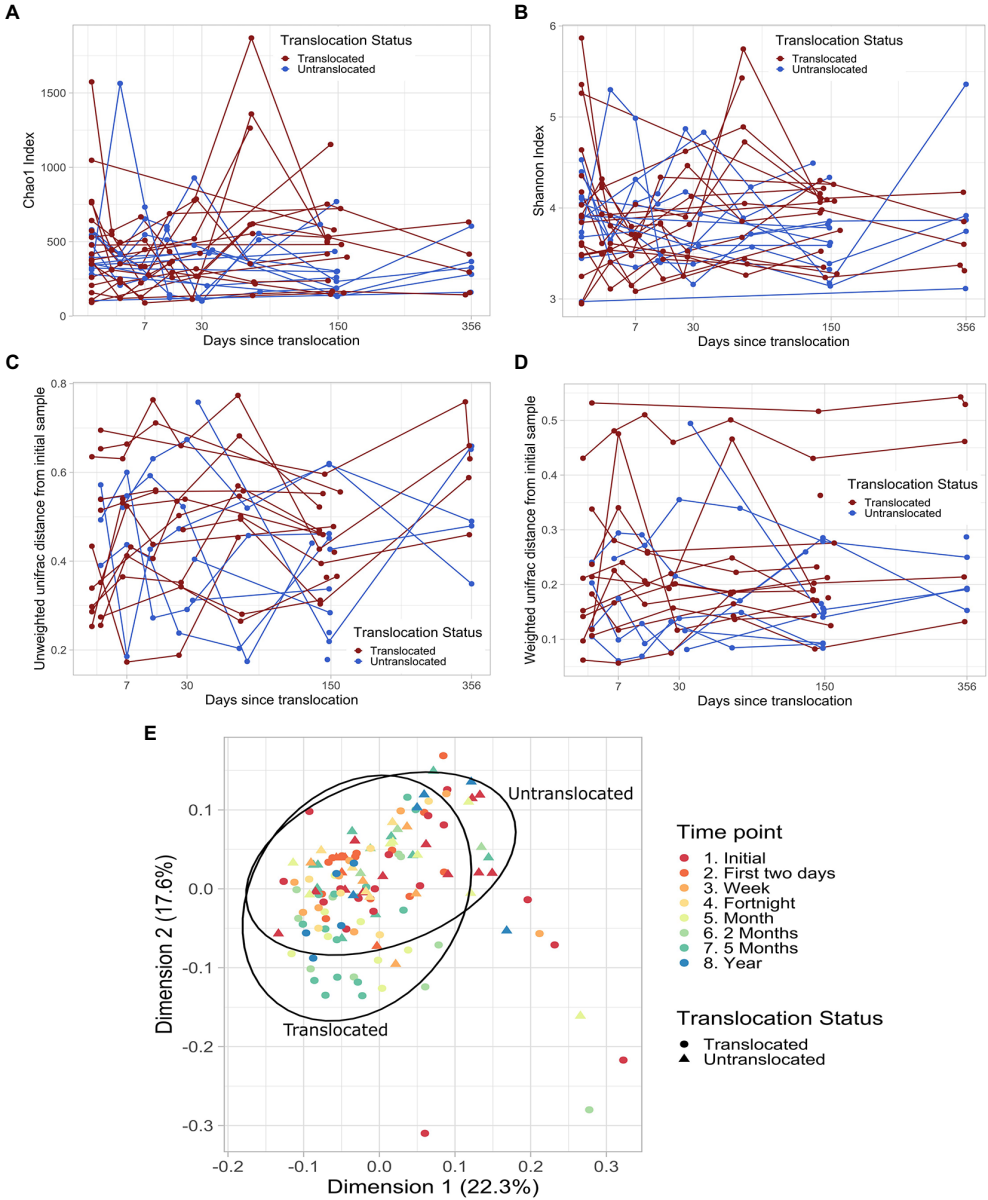
Fecal microbiome richness **(A)**, diversity **(B)** and composition **(C** and **D)** over time in individual translocated and untranslated koalas. **(A)** Chao1 diversity with days since translocation. Lines join sampling time points for the same koala. **(B)** Shannon diversity with days since translocation. Lines join sampling time points for the same koala. **(C)** Unweighted unifrac distance between each koala’s pre-translocation sample and subsequent samples against the days since translocation. Lines join sampling time points for the same koala. **(D)** Weighted unifrac distance between each koala’s pre-translocation sample and subsequent samples against the days since translocation. Lines join sampling time points for the same koala. **(E)** The first two principal components of the weighted unifrac distances for all koalas and time points in this study. 95% ellipses calculated assuming a multivariate t-distribution are shown for samples from translocated and untranslated koalas.

The richness and diversity of the koalas’ fecal microbiomes did not significantly differ between the translocated and untranslocated koala groups (Table 1). Nor did richness or diversity change in a consistent manner over time in either the translocated or control koala groups, whether the sampling time points were considered as discrete factors or as a continuous variable (Figures 1A,B).

**Table 1:** *p*-values from linear mixed effects models of the change in fecal microbiome richness, diversity, and composition in response to host translocation.

Response	Translocation status (TS)^1,2^	Time point (TP)^3^	TS × TP	Day^4^	TS × Day	Log(Day) (LD)^5^	TS × LD
Rank Feature count^6^	0.52	0.72	0.24	>0.99	0.46	0.68	0.11
Rank Chao1 Index^7^	0.58	0.76	0.29	0.91	0.56	0.67	0.14
Rank Shannon Index^8^	0.94	0.61	0.28	0.93	0.69	0.56	0.25
Weighted Unifrac^9,10^	0.15	0.87	0.72	0.61	0.78	0.82	0.23
Unweighted Unifrac^10,11^	0.23	0.44	0.12	0.49	0.44	0.90	0.06

Whether each koala was translocated or part of the control group of koalas left in the source habitat Type III p-values given for models including an interaction term with Time point. Backwards elimination was performed for each model to confirm the non-significance of all explanatory variables Samples were grouped into time points that were fitted as a factor Days since translocation were fitted as a continuous explanatory variable Days since translocation were log base 10 transformed and fitted as a continuous explanatory variable Rank Feature count = a measure of microbiome richness and is a count of the number of microbial features/sequence variants present in a sample, which is then rank transformed so that the model confirms to the assumption of normal residuals. Rank Chao1 Index = a measure of microbiome richness that estimates the number of microbial sequence variants present in a sample, correcting for undetected variants, which is then rank transformed so that the model confirms to the assumption of normal residuals. Rank Shannon Index = a measure of microbiome diversity, which is then rank transformed so that the model confirms to the assumption of normal residuals. Weighted Unifrac = a measure of how different two microbiomes are that is calculated based on the phylogenetic branch lengths between the taxa in each sample, weighted by the relative abundance of those taxa. In this analysis distances were calculated between each sampling period and the day 0 sample for each koala two outliers were removed from this analysis to meet the normality of residuals assumption. Unweighted Unifrac = a measure of how different two microbiomes are that is calculated based on the phylogenetic branch lengths between the taxa in each sample. In this analysis distances were calculated between each sampling period and the day 0 sample for each koala

Microbial community composition did not change in response to translocation when measured by weighted or unweighted unifrac distances, as there was no significant interaction between translocation status and any of the temporal measures (Table 1). Nor were the post-translocation microbiomes of the translocated koalas more dissimilar to their initial compositions than for control *in situ* koalas. There was no consistent temporal shift in the composition of the fecal microbiomes of either the translocated or control koalas (Figures 1C–E).

The microbiomes of the translocated koalas did not vary more between time points than those of the control koalas based on the unweighted or weighted unifrac distances between time points for each koala (unweighted unifrac: *p* = 0.57; weighted unifrac: *p* = 0.17).

**Figure 2:**
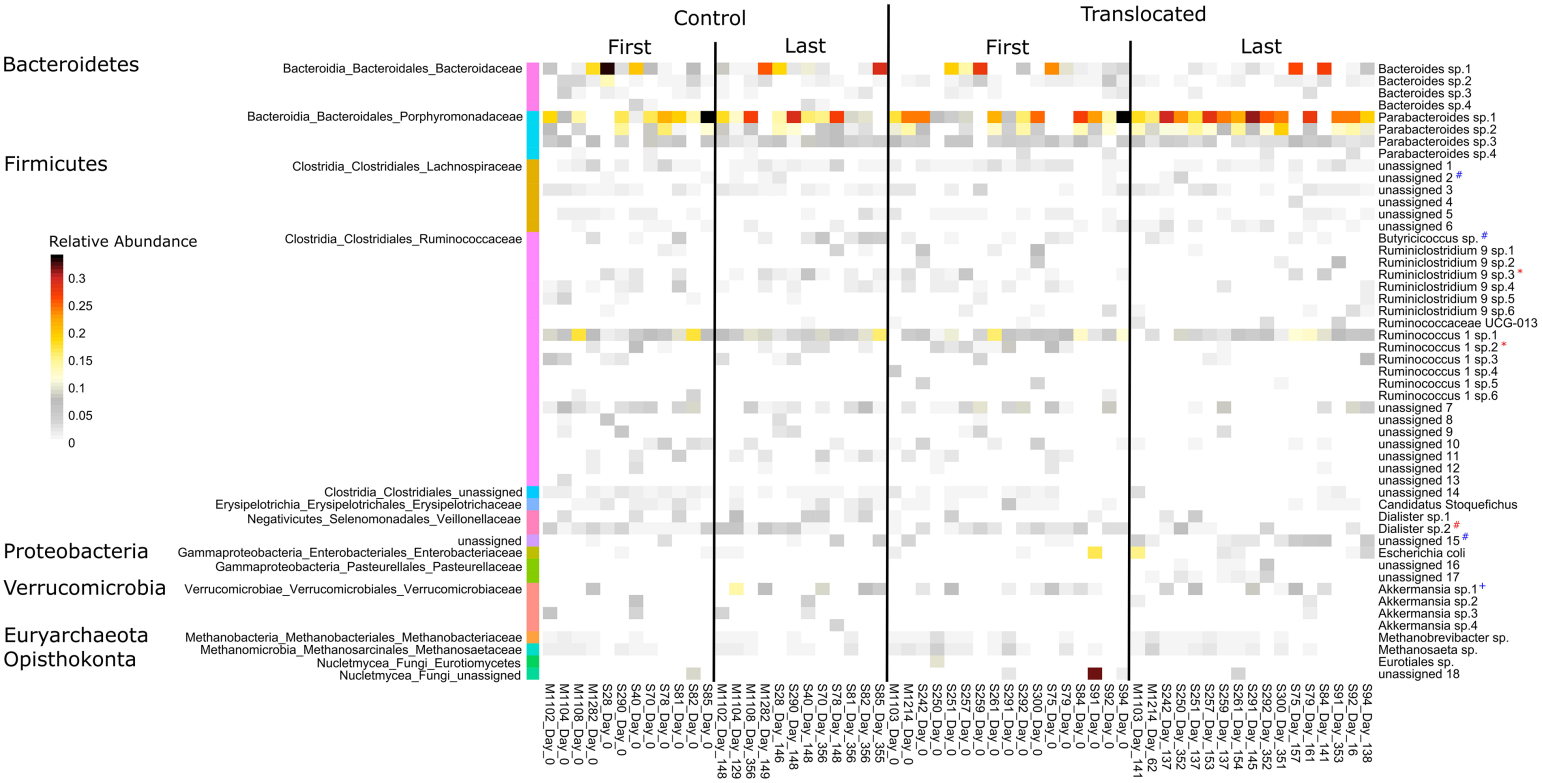
Heatmap showing the relative abundance of microbial features found at greater than 3% relative abundance in at least one sample for control (untranslocated) and translocated koalas prior to translocation (day 0) and on the last occasion where they were sampled. Species designations for the features are shown on the right while higher level taxonomy is shown on the left. # indicates features that significantly changed in relative abundance in both translocated and control koalas as identified by betabionomial mixed effects regression analysis. * indicates features that significantly changed in relative abundance only in the translocated koalas, while + indicates those that significantly changed only in control koalas. Blue symbols (#,+,*) indicate those features that significantly increased in relative abundance, while red symbols denote those that significantly decreased as identified by betabionomial mixed effects regression analysis.

Of the 132 prevalent features assessed (see methods), only 10 changed in relative abundance over the study, with a similar number of changes seen in each treatment group. Four features increased and two decreased in both the translocated and control koalas (rapid increase (log(Days)): 1 from phylum Synergistetes, genus *Cloacibacillus* and 2 unassigned shown in Figure 2; increase (Days): *Butyricicoccus* sp.; rapid decrease (log(Days)): Dialister sp.2; decrease (Days): 1 from phylum Firmicutes, family Lachnospiraceae). Additionally, Ruminococcaceae NK4A214 decreased in both the translocated and control koalas but to a greater extent in those that were translocated. *Akkermansia* sp.1 increased rapidly in the control koalas (log(Days)), while, *Ruminoccus*-1 sp.2 rapidly decreased in the translocated koalas (log(Days); Figure 2). *Ruminiclostridium* 9 sp.3 also decreased in the translocated koalas but not the control koalas. This concurs with the findings above, suggesting that host translocation did not lead to large changes in the koalas’ gut microbiomes.

### Effect of the gut microbiome on host condition

**Figure 3:**
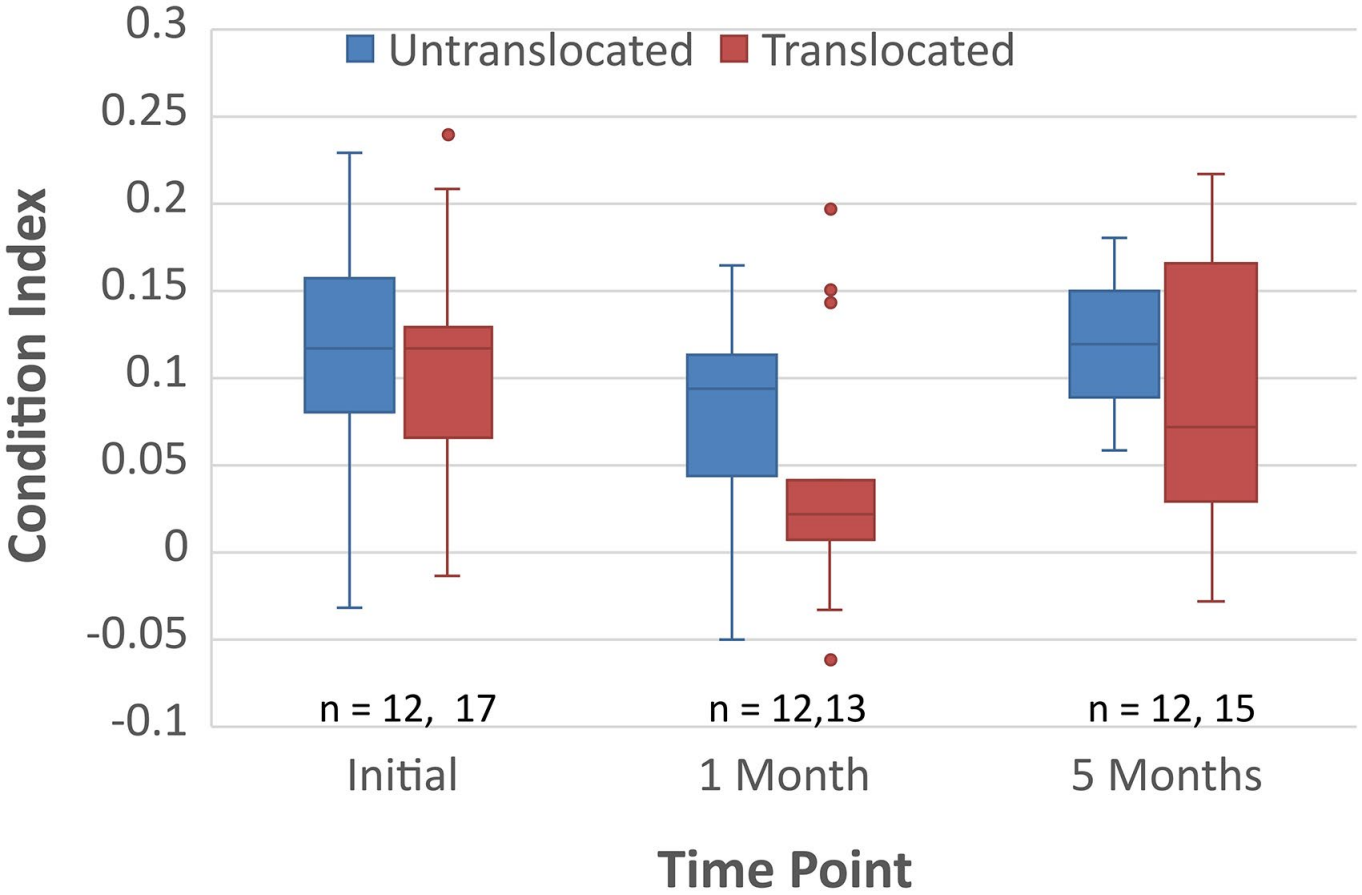
Body condition as determined by the method of McLean (2003) for the translocated and untranslocated koalas upon initial capture, 1 month post translocation and 5 months post translocation.

The translocated koalas in this study had a similar mortality rate to the control group, although, an initial drop in body condition was observed during the first month after translocation, followed by a recovery of condition by 5 months post translocation (Figure 3; Menkhorst et al., 2019). Therefore, we assessed whether the fecal microbiomes of the koalas prior to translocation affected their change in body condition 1 month post translocation.

**Figure 4:**
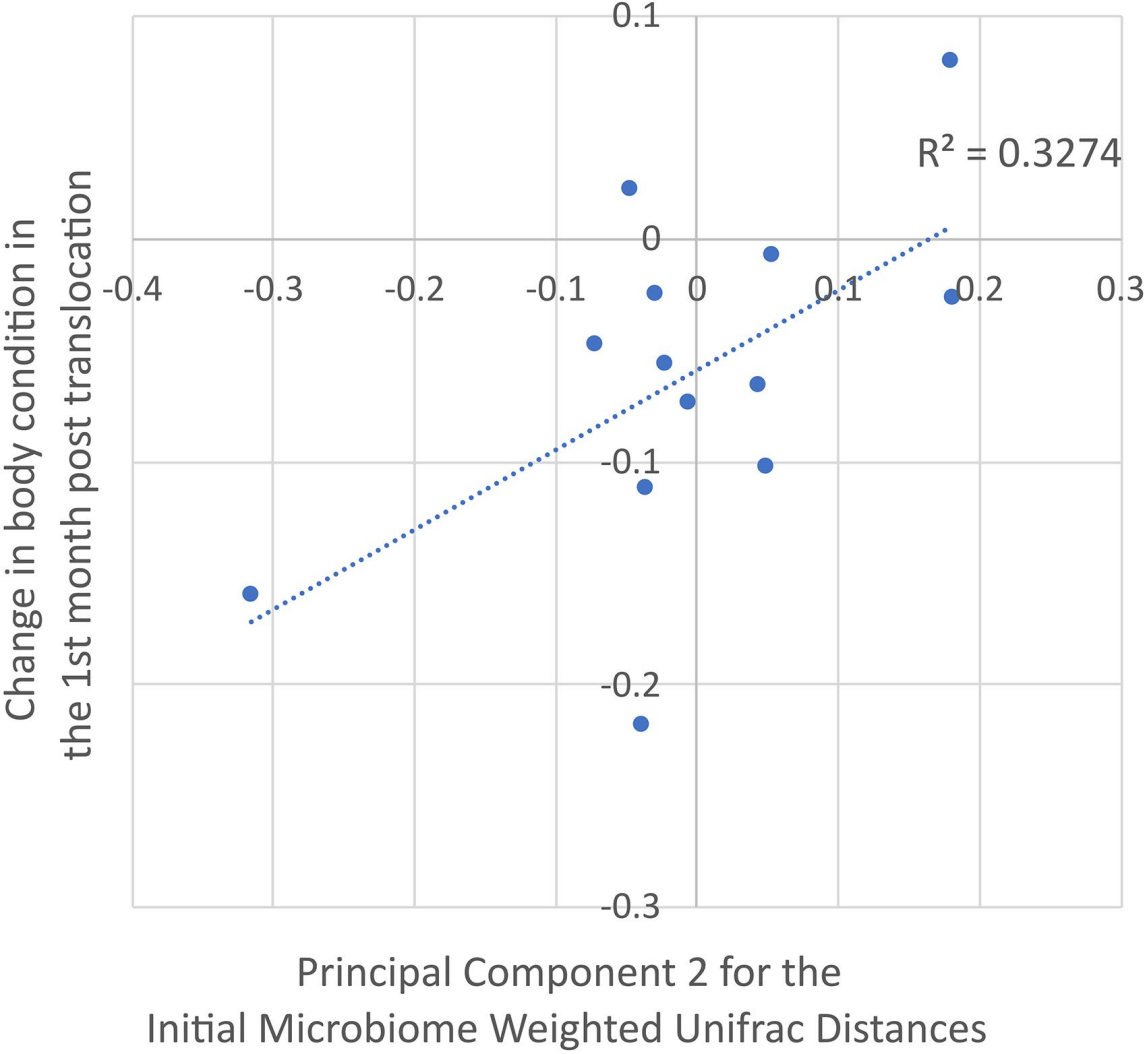
Correlation between dimension 2 of the principal components analysis of the translocated koalas’ weighted unifrac distances for their initial microbiomes (explaining 29.8% of the variation) and the change in their body condition over the first month post translocation.

Dimension 2 of the PCoA generated from the weighted unifrac distances among the translocated koalas’ initial microbiomes was a marginally significant predictor of their change in body condition over the first month post-translocation (R^2^ = 0.327, t = 2.31, *p* = 0.041; Figure 4). As the microbiome dimensions were generated from the weighted unifrac distances they cannot be directly related to any particular microbial taxon. However, the relative abundance of 27 of the 132 abundant microbial features had a greater than 20% positive correlation with Dimension 2 (Supplementary Table S1). These microbial features spanned a range of taxa including the phyla Euryarchaeota, Actinobacteria, Bacteroidetes, Chloroflexi, Firmicutes, Planctomycetes and Proteobacteria with no strong phylogenetic signal observable. None of the other dimensions from the PCoAs of the unweighted and weighted distances among the koalas’ initial microbiomes nor initial microbiome richness or diversity were significant predictors of the change in body condition over the first month post-translocation.

No measures of diet composition post translocation were significant predictors of the change in body condition over the first month post translocation.

### Effect of the gut microbiome on dietary choice

**Figure 5:**
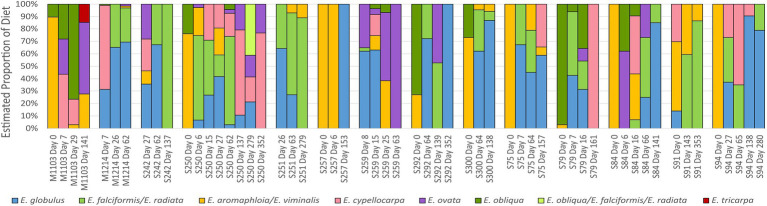
Diet composition for the translocated koalas as determined by selectively amplifying and sequencing species-specific SNPs from dietary food tree species using the DarTag platform (Diversity Arrays Technologies). The SNPs were amplified from DNA extracted from faecal pellets in the same collections that were used for microbiome assessment in this study. Day 0 samples were collected from the koalas prior to translocation, while all other samples were collected at the indicated days after translocation. S and M numbers correspond to individual koala IDs. Figure reproduced from (Blyton et al., 2023).

The diets of the translocated koalas were assessed prior to and after translocation from fecal pellets using a newly developed panel of species-specific SNPs (Blyton et al., 2023). This analysis revealed that the koalas’ diets rapidly changed after translocation (Figure 5). Pre-translocation, *E. viminalis* dominated the diets of the majority of koalas, while *E. obliqua* was also eaten by half the koalas. After translocation, the koala diets became more species-rich and variable among individuals with *Eucalyptus globulus*, *Eucalyptus cypellocarpa*, and *Eucalyptus radiata* most often the dominant components.

**Figure 6:**
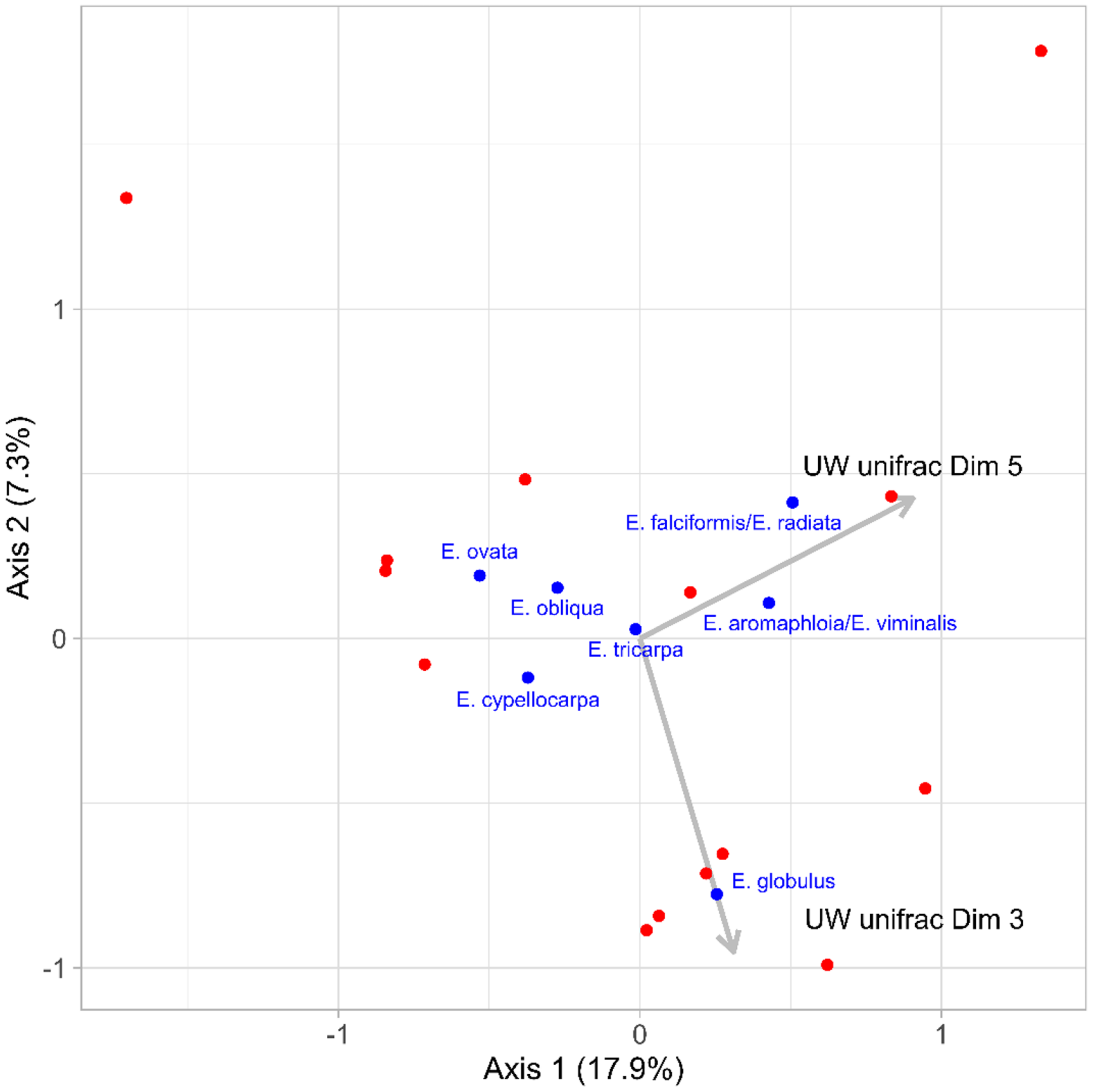
Constrained dimensions from the redundancy analysis of the translocated koalas’ diets post-translocation (red points). Dimensions 3 and 5 from the principal components analysis of the unweighted unifrac distances of the koalas’ initial microbiomes were significant predictors of the koalas’ post translocation diets. The loadings for Dimensions 3 and 5 are illustrated by the gray arrows. The loadings of the dietary food tree species are indicated by the blue points and font.

Redundancy analysis revealed that the microbiomes of koalas prior to translocation (day 0) influenced what species of eucalypt they ate in the new habitat. Both dimensions 3 and 5 of the PCoA generated from the unweighted unifrac distances of the translocated koalas’ initial microbiomes, were significant predictors of the post-translocation diets of koalas (Dimension 3: *F* = 2.72, *p* = 0.03; Dimension 5: *F* = 2.61, p = 0.03). Dimension 3 was associated with diets containing a high proportion of *E. globulus* and Dimension 5 was associated with diets containing a high proportion of *Eucalyptus falciformis/E. radiata* and to a lesser extent *Eucalyptus aromaphloia/E. viminalis* (Figure 6). Together, these two dimensions of microbiome variation accounted for 37.1% of the variation in diet. As the microbiome dimensions were generated from the unweighted unifrac distances they cannot be directly related to specific microbial taxa. However, there was a negative correlation between the number of microbial features belonging to the families Bacteroidaceae (range: 3–10 features; *R*^2^ = 0.129) and Veillonellaceae (range: 0–4 features; *R*^2^ = 0.445) and Dimension 3. The number of microbial features belonging to the families Lachnospiraceae (range: 9–42 features; *R*^2^ = 0.210), Rikenellaceae (range: 1–3 features; *R*^2^ = 0.292) and Ruminococcaceae (range: 6–23 features; *R*^2^ = 0.127) were negatively corelated with Dimension 5.

There was no association between the diversity of the koalas’ microbiomes at the time of translocation and the diversity of their diets in the destination habitat; either in terms of the number of tree species eaten (Chao1 index: *p* = 0.32; Shannon microbial diversity index: *p* = 0.31) or Shannon diet diversity index (Chao1 index: *p* = 0.48; Shannon microbial diversity index: *p* = 0.29).

### Association between diet and microbiome variation

**Figure 7:**
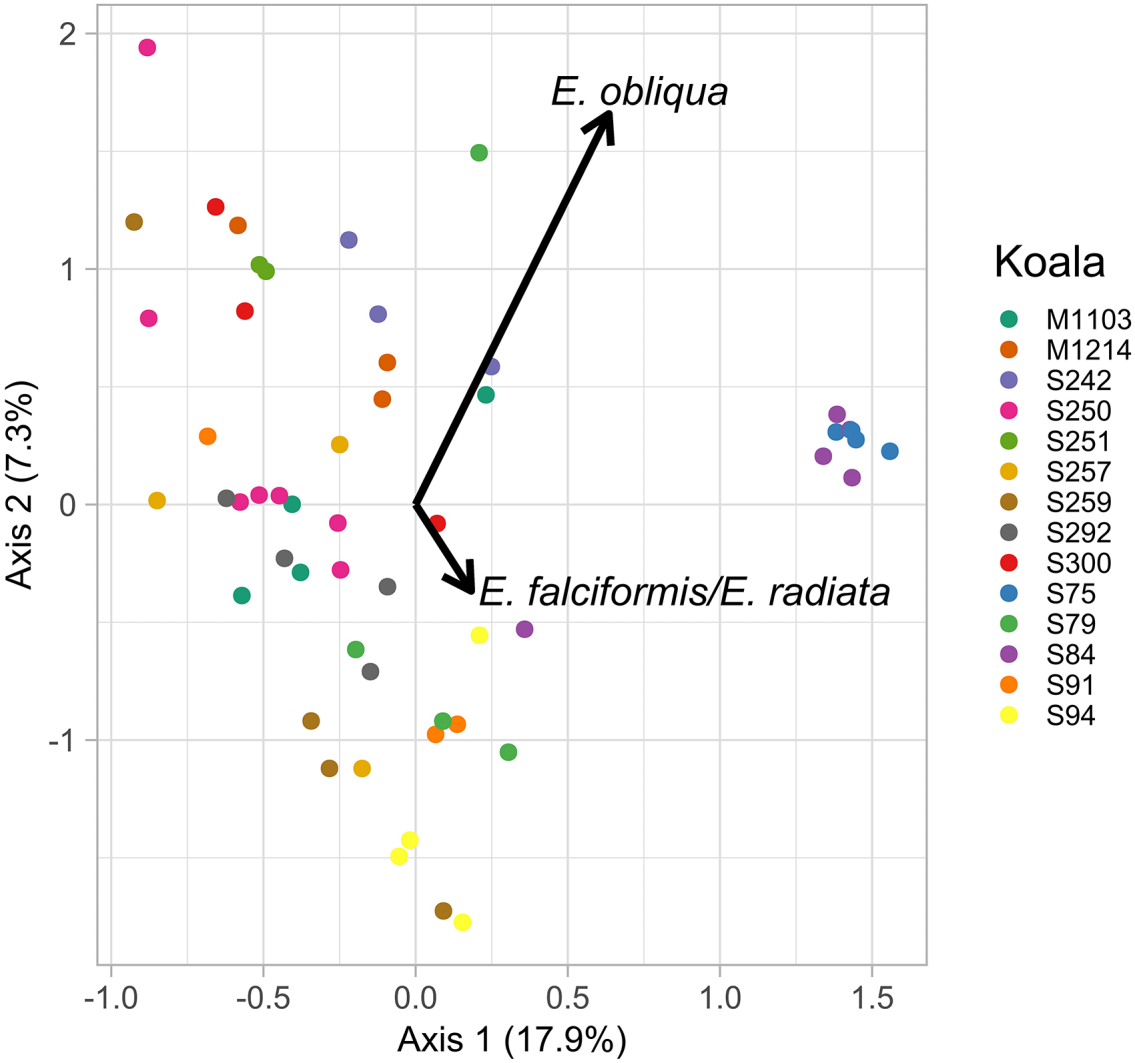
Constrained dimensions one and two from the redundancy analysis of the translocated koalas’ faecal microbiomes at each sampling time point (colored points). The proportions of *E. obliqua* and *E. radiata* detected in the samples at each time point were significant predictors of microbiome composition and their loadings are illustrated by the black text and arrows.

Although there was not a consistent directional change in the fecal microbiomes of translocated koalas in this study, variation in microbiome composition was nonetheless observed for individual koalas between sampling time points. Therefore, in an explorative analysis we investigated whether temporal variation in the microbiomes of translocated koalas could in part be attributed to changes in the diets of these animals. Redundancy analysis revealed that the proportion of *E. obliqua* (*p* = 0.03) and *E. falciformis/E. radiata* (*p* = 0.04) eaten at a particular time point was associated with the relative abundances of the microbial features in the koalas’ feces at that time (Figure 7). However, while differences between koalas accounted for 52.9% of the microbiome variation, the proportion of *E. obliqua* and *E. radiata* together only accounted for 5% of the microbiome variation.

## Discussion

Across a range of animal species, including humans, the gut microbiome has been found to change and adapt to variation in host diet (Hungate, 1966; David et al., 2014; Xu and Knight, 2015; Barron Pastor and Gordon, 2016). However, in hindgut fermenting herbivores such as the koala where much of the nutritious portion of the diet is absorbed in the small intestine prior to entering the caecum (Cork et al., 1999), the effect of diet on the hindgut and fecal microbiome may be more restricted. In this study the fecal microbiomes of translocated koalas did not change compared to those of koalas that remained in their original habitat. This was despite diversification of the translocated koalas’ diets and a change in the species of *Eucalyptus* eaten (Blyton et al., 2023). One explanation for the observed stability of the koala microbiome could be that the change in diet may have represented only a subtle shift in the nutritional composition of the koalas’ diets when compared with the large changes examined in other species. However, this explanation is unlikely, as we and others have previously shown that koala food tree species can differ markedly in their nutritional (Petrović, 2014; Brice et al., 2019) and plant secondary metabolite composition (Moore et al., 2005; Marsh et al., 2019). Furthermore, multiple *Eucalyptus* subgenera were eaten by koalas in this study and compositional differences among food tree species are most apparent between species in different subgenera. In this instance, the variation in diet between individuals after translocation and the finding that the koalas’ pre-translocation microbiomes were associated with post translocation diet may indicate that the koalas were able to find diets in the destination habitat that suited their existing microbiomes, limiting the extent to which adaptation of their microbiomes were necessary. However, in our previous work, the gut microbiomes of koalas were found to be unaffected by an experimentally induced change in diet to which their microbiomes were not adapted (Blyton et al., 2019). Therefore, the most credible explanation for the lack of change in the microbiomes of the translocated koalas is that they had a limited ability to respond and adapt to dietary change.

Koalas maternally inherit their gut microbiomes through the ingestion of a special feces (pap) around the time of pouch emergence and prior to the joeys’ transition from a milk-based diet onto a diet of *Eucalyptus* leaves (Osawa et al., 1993; Blyton et al., 2022a, 2022b). This appears to lead to varying, but individual koala microbiomes that are temporally stable (Eisenhofer et al., 2022). Eisenhofer et al. (2022) found that captive koalas sourced from different populations but fed on a similar diet maintained distinct microbiomes. In contrast, wild koalas in a single population, feeding on diverse diets, had more similar microbiomes. Indeed, in the present study we found that while temporal variation in diet led to small changes in the translocated koalas’ fecal microbiomes, the majority of variation could be attributed to interindividual differences observed prior to translocation that were maintained. Further, diet was only associated with microbial community composition measures that are heavily influenced by the relative abundance of the different microbial species (weighted unifrac distances) suggesting that diet does not alter the presence/absence of microbes. These studies suggest that the gut microbiomes of koalas are primarily determined by their acquisition and development during early life, with subsequent diet having a comparatively weak effect.

While koala gut microbiomes do not readily change with alterations in diet, the fecal microbiomes of koalas with consistently different diets are distinct (Brice et al., 2019). In other species, the gut microbiome has been shown to affect diet selection *via* toxins in the hosts’ diet that can be broken down by particular microbial species. For instance, Australian cattle can only eat *Leucaena leucocephala* when bacteria that degrade mimosine are introduced into their rumen (Pratchett et al., 1991; Derakhshani et al., 2016). Additionally, when the foregut pouches of woodrats (*Neotoma* spp.) are inoculated with oxalate degrading bacteria from woodrats eating juniper or oxalate-rich cactus species they are able to maintain condition on those diets, where prior to inoculation they ate little and lost weight (Kohl et al., 2014; Miller et al., 2014). Previously, we experimentally altered the microbiomes of wild caught koalas using fecal transplants and showed that koalas with greater engraftment consumed more of an unfamiliar *Eucalyptus* species that formed the dominant component of the fecal donors’ diets. This suggests that the gut microbiome may also influence feeding choices of koalas (Blyton et al., 2019). However, one limitation of our earlier study was that, as a group, the koalas that received fecal transplants did not consume more of the novel *Eucalyptus* species than control koalas, reducing certainty of the causality of the observed diet-microbiome association.

The translocated koalas in this study maintained relatively stable microbiomes over time and post translocation the koalas’ diets changed in response to the availability of new food tree species but with individual koalas selecting different mixes of species. Host factors, such as physiological variation between individuals in their ability to detoxify particular plant secondary metabolites (McLean and Duncan, 2006), likely influenced these dietary choices. Variation in the food tree species within the translocated koalas’ home ranges also likely influenced their diets, although the translocated koalas initially moved quite large distances and those movements may have been driven in part by diet selection (Menkhorst et al., 2019). Additionally, our finding that the initial fecal microbiomes of the translocated koalas were significant predictors of the koalas’ post-translocation diets, accounting for over a third of the variation in diet, provides compelling evidence that the gut microbiome does indeed influence diet selection in koalas. In our fecal transplant experiment, *E. obliqua* intake declined over the first 3 days post-introduction, which we suggested may have been due to the koalas developing an aversion to the new species through post-ingestive feedback (Provenza et al., 1992; Lawler et al., 1999). Conceivably, the microbiomes of the koalas in this study may have influenced dietary choice in the destination habitat in a similar way. That is the koalas’ gut microbiomes may have influenced post-ingestive feedback as a result of their identified effects on digestion, nutrition, and detoxification (Cork and Hume, 1983; Osawa, 1992; Osawa et al., 1995).

Although the composition of the koalas’ gut microbiomes may have influenced their diet in the destination habitat, we still lack an adequate understanding of the functional significance of particular microbial taxa, on which these associations with particular eucalypt species are based. In this study, we detected the strongest microbiome associations with diets dominated by either *E. globulus* or *E. falciformis*/*E. radiata*. The genetic method used to identify the species of *Eucalyptus* eaten by the koalas in this study was not able to differentiate *E. falciformis* from *E. radiata.* However, *E. falciformis* was rare in the home ranges of the translocated koalas and it is therefore likely that the koalas were not feeding on that species and only on *E. radiata* (Blyton et al., 2023). *Eucalyptus radiata* is often avoided by koalas (Martin, 1985c) and is less digestible than *E. globulus* because it contains more fiber and higher concentrations of tannins (Foley and Hume, 1987; Petrović, 2014). *E. globulus,* by contrast, is a highly used koala food tree (Hynes et al., 2021) and provides relatively digestible foliage that likely provides more metabolizable energy to koalas, in conjunction with high nitrogen (protein) availability (Petrović, 2014). In this study, the Firmicutes family Veillonellaceae was negatively associated with a diet dominated by *E. globulus* and the families Lachnospiraceae and Ruminococcaceae were negatively associated with *E. radiata.* In previous studies, we have identified a positive association between the relative abundance of the phylum Firmicutes, particularly members of the families Lachnospiraceae and Ruminococcaceae, and the consumption of *E. obliqua*. *Eucalyptus obliqua* is high in fiber and low in available nitrogen compared with the dominant food tree species, *E. viminalis,* consumed by that koala population (Blyton et al., 2019; Brice et al., 2019). While *E. radiata* may appear nutritionally similar to *E. obliqua*, and *E. globulus* nutritionally similar to *E. viminalis*, species-specific differences may account for the differing microbial associations observed in this study. Further, there is substantial functional variation among members of the same microbial family (Biddle et al., 2013). Thus, more detailed functional studies at a finer taxonomic resolution are required to elucidate these microbiome-nutrition associations. Notably though, only the unweighted unifrac distances were associated with diet composition in this study, suggesting that it may be the presence of particular microbial species rather than their relative abundances that is important to diet selection. As such, it is possible that our previous relative abundance findings may reflect underlying differences in the microbial species.

The gut microbiomes were inferred to influence not only the diets of koalas, but also their condition during the first month post-translocation. However, the associated microbiome characteristics were different in each case. Further, there was no evidence that post translocation diets influenced koala condition. This suggests that the observed association between the koalas’ body condition and the initial fecal microbiomes were not linked to their diet. Instead, it is conceivable that the koalas’ microbiomes were associated with other aspects of the koalas’ physiology and/or health. While not directly applicable to this study, koalas suffering from chlamydial infection and those that die from antibiotic treatment appear to have gut microbiomes that differ from those of healthy individuals, with koalas that recover from antibiotic treatment regaining a microbiome community similar to that of healthy koalas (Barker et al., 2013; Alfano et al., 2015; Dahlhausen et al., 2018). Additionally, in our study of gut microbiome development in captive joeys we observed that two captive koalas that later died from non-infectious diseases had dissimilar gut microbiomes to those of other adult koalas in that study and that they passed those distinct microbiomes onto their joeys (Blyton et al., 2022b). Further studies are needed to determine if the gut microbiome could be used as a marker for host health in koalas. Establishing whether the abnormal microbiomes of sick koalas contribute to their condition or are merely a symptom of underlying pathology should also be an important area of further research.

Overall, there were few changes in the relative abundance of particular microbial species in either the translocated or control koalas. However, one species, *Akkermansia* sp.1, is of interest with regard to koala health. Members of the genus *Akkermanisa* have been found to increase in relative abundance in fasted Burmese pythons and Syrian hamsters (Sonoyama et al., 2009; Costello et al., 2010). In humans, *Akkermanisa muciniphila* has been shown to feed on host mucin, allowing it to survive when nutrients become limiting such as during host starvation (Derrien et al., 2004). Captive and wild adult koalas have been reported to carry *Akkermanisa* at an average relative abundance of 1.5%–3.1% and 2.2%, respectively (Eisenhofer et al., 2022; Blyton et al., 2022a, 2022b). In this study, *Akkermansia* increased in relative abundance in the control koalas from an average of 2% to 4.9% in June 2016. Interestingly, koalas feeding on mana gum at Cape Otway (the site of this study) had *Akkermanisa* relative abundances of 1.0% in 2015 (prior to this study), and 3.4% in 2017–2018 (a year after the completion of this study) (Blyton et al., 2019; Brice et al., 2019). Thus, the high relative abundance of *Akkermanisa* observed in the control koalas in this study could indicate that they suffered from some level of starvation. In line with this, Menkhorst et al. (2019) observed that males in the control group of this study had a continued decline in body condition and suggested that this was due to the defoliated habitat at the source site. Notably, however, in 2013 when defoliation and starvation was at its peak at the site, the average relative abundance of *Akkermanisa* was 2.0% in koalas feeding on manna gum (Whisson et al., 2016; Brice et al., 2019). Although, it is not known if the particular koalas sampled at that time were starving.

The findings from our analysis of the microbiomes and diet of the koalas in this successful translocation project provide several insights for improving future koala translocation efforts. As the koala gut microbiome appears to be largely unaffected by dietary changes, we suggest that the *Eucalyptus* species at the destination habitat be matched to those in the source habitat wherever possible. Alternatively, where food tree species cannot be matched between the source and destination habitats, we suggest selecting a destination site that has a range of different food tree species as was done in this study. Mortality at the destination habitat in this study was not greater than at the source habitat over the same period (Menkhorst et al., 2019). The translocated koalas had access to at least 10 species of *Eucalyptus* in the destination habitat and at least six to seven of these were food tree species (Figure 5). Koalas can thrive on a single food tree species where that species has high nutritional quality (Ashman et al., 2020; Whisson and Ashman, 2020). Prior to translocation the koalas’ diets were near monophagous for one of these preferred species; *E. viminalis.* After translocation, the koalas developed individual differences in their diets and there was an association between their post-translocation diets and starting microbiomes. Together these findings suggest that a landscape containing multiple different food tree species from which the koalas could select their diets after translocation may have allowed them to find species to which their microbiomes were suited, contributing to their high survival rates. While careful site selection is essential to the success of any translocation, in some cases it may not be possible to locate destination sites with a range of food tree species. In such cases, the finding from this study and our previous work that the koala microbiome remains stable despite diet changes indicates that fecal inoculations (Blyton et al., 2019) could be useful for adapting their microbiomes to a small number of unfamiliar food tree species in a destination habitat.

## Conclusion

This study provides evidence that microbiome-diet associations in the koala are more convincingly explained by the influence of gut microbiome composition on diet selection than the reverse. Our findings also indicate that the koala gut microbiome is largely unperturbed by diet change, which in some instances could be to the detriment of the animal. This suggests that the success of koala translocations would be enhanced by ensuring that food tree availability in destination habitats matches that of the source. If the exact vegetation associations are not available, then ensuring a variety of food tree species are available will increase the likelihood of a microbiome-diet match. Additionally, the association between the koalas’ initial microbiome and their post-translocation condition suggests that further research is needed to establish the role of the koala gut microbiome in host health. The stability of the koala’s gut microbiome despite major upheaval is in contrast to the plasticity seen in some other species and demonstrates that the response of the gut microbiome to translocation is likely to be host species-specific.

## Data availability statement

The data presented in the study are deposited in the NCBI SRA repository, Bioproject accession number PRJNA901215.

## Ethics statement

The animal study was reviewed and approved by the Western Sydney University Animal Care and Ethics Committee. Fieldwork was carried out under the approval of the Western Sydney University Animal Care and Ethics Committee (A11253) and with an appropriate permit from the Victorian government (10007714).

## Author contributions

MB and BM designed the project. MB, JP, and EH completed the fieldwork including fecal sample collection. RS performed the DNA extractions and sequencing. MB performed the statistical analyses and wrote the first draft of the manuscript. All authors contributed to the article and approved the submitted version.

## Funding

This research was funded by the Australian Research Council (LP140100751) in partnership with Evolva Biotech A/S, the NSW Office of Environment and Heritage and The Conservation Ecology Trust (Cape Otway) and in part by an ARC Discovery Project (DP150104202).

## Conflict of interest

The authors declare that the research was conducted in the absence of any commercial or financial relationships that could be construed as a potential conflict of interest.

## Publisher’s note

All claims expressed in this article are solely those of the authors and do not necessarily represent those of their affiliated organizations, or those of the publisher, the editors and the reviewers. Any product that may be evaluated in this article, or claim that may be made by its manufacturer, is not guaranteed or endorsed by the publisher.
