# Supplementary material for: The koala gut microbiome is largely unaffected by host translocation but rather influences host diet
Source: Front Microbiol. 2023 Mar 2;14:1085090. doi: 10.3389/fmicb.2023.1085090 (PMC10018171; doi:10.3389/fmicb.2023.1085090)
Supplement: Supplementary file 1 [file Table_1.DOCX]

**Table S1: Microbial features whose relative abundances were positively correlated (R^2^ > 20%) with Dimension 2 of the PCoA of the weighted unifac distances**

| R^2^ | Taxonomy |
| --- | --- |
| 0.45 | Archaea;Euryarchaeota;Methanobacteria;Methanobacteriales;Methanobacteriaceae;Methanobrevibacter |
| 0.35 | Archaea;Euryarchaeota;Methanomicrobia;Methanosarcinales;GOM Arc I;Candidatus Methanoperedens;uncultured archaeon |
| 0.32 | Archaea;Euryarchaeota;Methanomicrobia;Methanosarcinales;Methanosaetaceae;Methanosaeta;uncultured archaeon |
| 0.47 | Bacteria;Actinobacteria;Actinobacteria;Corynebacteriales;Corynebacteriaceae;Corynebacterium 1 |
| 0.27 | Bacteria;Actinobacteria;Actinobacteria;Corynebacteriales;Corynebacteriaceae;Corynebacterium 1 |
| 0.36 | Bacteria;Actinobacteria;Actinobacteria;Corynebacteriales;Corynebacteriaceae;Corynebacterium 1;uncultured bacterium |
| 0.33 | Bacteria;Bacteroidetes;Bacteroidia;Bacteroidales;Bacteroidaceae;Bacteroides;uncultured bacterium |
| 0.39 | Bacteria;Bacteroidetes;Bacteroidia;Bacteroidales;Bacteroidaceae;Bacteroides;uncultured bacterium |
| 0.46 | Bacteria;Bacteroidetes;Bacteroidia;Bacteroidales;Bacteroidales S24-7 group;uncultured bacterium;uncultured bacterium |
| 0.34 | Bacteria;Bacteroidetes;Bacteroidia;Bacteroidales;Porphyromonadaceae |
| 0.54 | Bacteria;Bacteroidetes;Bacteroidia;Bacteroidales;Prevotellaceae |
| 0.52 | Bacteria;Bacteroidetes;Bacteroidia;Bacteroidia Incertae Sedis;Draconibacteriaceae;uncultured;uncultured bacterium |
| 0.26 | Bacteria;Bacteroidetes;Sphingobacteriia;Sphingobacteriales;Saprospiraceae;uncultured;uncultured bacterium |
| 0.37 | Bacteria;Chloroflexi;Anaerolineae;Anaerolineales;Anaerolineaceae |
| 0.27 | Bacteria;Chloroflexi;Anaerolineae;Anaerolineales;Anaerolineaceae;uncultured |
| 0.44 | Bacteria;Firmicutes;Bacilli;Lactobacillales;Aerococcaceae;Facklamia;uncultured bacterium |
| 0.39 | Bacteria;Firmicutes;Bacilli;Lactobacillales;Carnobacteriaceae |
| 0.33 | Bacteria;Firmicutes;Bacilli;Lactobacillales;Carnobacteriaceae;Atopostipes;uncultured bacterium |
| 0.42 | Bacteria;Firmicutes;Bacilli;Lactobacillales;Streptococcaceae;Streptococcus |
| 0.35 | Bacteria;Firmicutes;Clostridia;Clostridiales;Clostridiaceae 1;Clostridium sensu stricto 1 |
| 0.28 | Bacteria;Firmicutes;Erysipelotrichia;Erysipelotrichales;Erysipelotrichaceae;Turicibacter;uncultured bacterium |
| 0.47 | Bacteria;Planctomycetes;Planctomycetacia;Planctomycetales;Planctomycetaceae;p-1088-a5 gut group;uncultured bacterium |
| 0.31 | Bacteria;Proteobacteria;Betaproteobacteria;Rhodocyclales;Rhodocyclaceae |
| 0.32 | Bacteria;Proteobacteria;Deltaproteobacteria;Syntrophobacterales;Syntrophaceae;Smithella;uncultured bacterium |
| 0.32 | Bacteria;Proteobacteria;Gammaproteobacteria;Pseudomonadales;Moraxellaceae;Psychrobacter |
| 0.54 | Bacteria;Proteobacteria;Gammaproteobacteria;Xanthomonadales;Xanthomonadales Incertae Sedis;Candidatus Competibacter |
| 0.26 | Unassigned |
